# Real-world patterns of opioid therapy initiation in Spain, 2012–2018: A population-based, retrospective cohort study with 957,080 patients and 1,509,488 initiations

**DOI:** 10.3389/fphar.2022.1025340

**Published:** 2022-11-16

**Authors:** Isabel Hurtado, Celia Robles, Salvador Peiró, Aníbal García-Sempere, Fran Llopis-Cardona, Francisco Sánchez-Sáez, Clara Rodríguez-Bernal, Gabriel Sanfélix-Gimeno

**Affiliations:** ^1^ Health Services Research Unit, Foundation for the Promotion of Health and Biomedical Research of Valencia Region, Valencia, Fisabio; ^2^ Network for Research on Chronicity Primary Care and Health Promotion (RICAPPS), Valencia, Fisabio

**Keywords:** opioids, non-cancer pain, patterns of initiation, population-based study, patient characteristics

## Abstract

**Introduction:** Europe has seen a steady increase in the use of prescription opioids, especially in non-cancer indications. Epidemiological data on the patterns of use of opioids is required to optimize prescription. We aim to describe the patterns of opioid therapy initiation for non-cancer pain and characteristics of patients treated in a region with five million inhabitants in the period 2012 to 2018.

**Methods:** Population-based retrospective cohort study of all adult patients initiating opioid therapy for non-cancer pain in the region of Valencia. We described patient characteristics at baseline and the characteristics of baseline and subsequent treatment initiation. We used multinominal regression models to identify individual factors associated with initiation.

**Results:** A total of 957,080 patients initiated 1,509,488 opioid treatments (957,080 baseline initiations, 552,408 subsequent initiations). For baseline initiations, 738,749 were with tramadol (77.19%), 157,098 with codeine (16.41%) 58,436 (6.11%) with long-acting opioids, 1,518 (0.16%) with short-acting opioids and 1,279 (0.13%) with ultrafast drugs. When compared to tramadol, patients initiating with short-acting, long-acting and ultrafast opioids were more likely to be older and had more comorbidities, whereas initiators with codeine were more prone to be healthier and younger. Treatments lasting less than 7 days accounted for 41.82% of initiations, and 11.89% lasted more than 30 days. 19.55% of initiators with ultrafast fentanyl received more than 120 daily Morphine Milligram Equivalents (MME), and 16.12% of patients initiating with long-acting opioids were prescribed more than 90 daily MME (*p* < 0.001). Musculoskeletal indications accounted for 65.05% of opioid use. Overlap with benzodiazepines was observed in 24.73% of initiations, overlap with gabapentinoids was present in 11.04% of initiations with long-acting opioids and 28.39% of initiators with short-acting opioids used antipsychotics concomitantly. In subsequent initiations, 55.48% of treatments included three or more prescriptions (vs. 17.60% in baseline initiations) and risk of overlap was also increased.

**Conclusion:** Opioids are initiated for a vast array of non-oncological indications, and, despite clinical guidelines, short-acting opioids are used marginally, and a significant number of patients is exposed to potentially high-risk patterns of initiation, such as treatments lasting more than 14 days, treatments surpassing 50 daily MMEs, initiating with long-acting opioids, or hazardous overlapping with other therapies.

## Introduction

Over the past 20 years, Europe has seen a steady increase in the use of prescription opioids. Even if the region is not at risk of an opioid crisis as seen in the US in the 2000s, several studies have raised concerns about growing trends of prescription opioid use, especially in non-cancer indications, and the lack of robust epidemiological data required to optimize opioid prescription and to adequately address and prevent potential harms (Morgan et al., 2008; van Amsterdam and van den Brink, 2015; Helmerhorst et al., 2017; Verhamme Bohnen, 2019; Alho et al., 2020; Häuser et al., 2021; Seyler et al., 2021; van Amsterdam et al., 2021; Xie et al., 2022).

Spain is no exception and the volume of opioid prescription, the number of patients treated, and intensity of treatment in terms of Morphine Milligram Equivalents (MME) have dramatically increased (Xie et al., 2022; Hurtado et al., 2020; Garcia del Pozo et al., 2008; Ruiz-López and Alonso-Babarro, 2019; Oliva et al., 2022; Spanish Observatory, 1144). Moreover, additional concerns about opioid treatment effectiveness in clinical practice, their present and future associated cost burden, or the presence of potential patterns of misuse have been raised (Spanish Observatory, 1144; Sicras-Mainar et al., 2021; Rejas-Gutierrez et al., 2021; Fonseca et al., 2017), therefore stressing the need of gaining a better understanding of the patterns of use of the different types of opioids in the population.

In Valencia, a region with five million inhabitants, a previous study showed that the yearly volume of opioid prescription doubled during the period 2010–2018 (Hurtado et al., 2020), led by a high volume, high growth prescription of tramadol, and prescribed doses in terms of Morphine Milligram Equivalents (MME) per capita showed almost a threefold increase, due to the notable growth of strong opioid use (fentanyl, oxycodone and tapentadol). During the referred period, the annual number of patients receiving at least one opioid prescription more than doubled (Hurtado et al., 2020). These concerning trends suggest that opioid use in non-cancer pain may be widespread and warrants investigation about the underlying patterns of utilisation of opioids overall and per type of opioid. In this study we aim to describe the patterns of opioid therapy initiation for non-cancer pain in our setting and the characteristics of patients treated, as well as to identify factors associated with opioid therapy initiation.

## Methods

### Design

Population-based retrospective cohort study of all adult patients initiating opioid therapy for non-cancer pain in the region of Valencia.

### Setting and population

**FIGURE 1:**
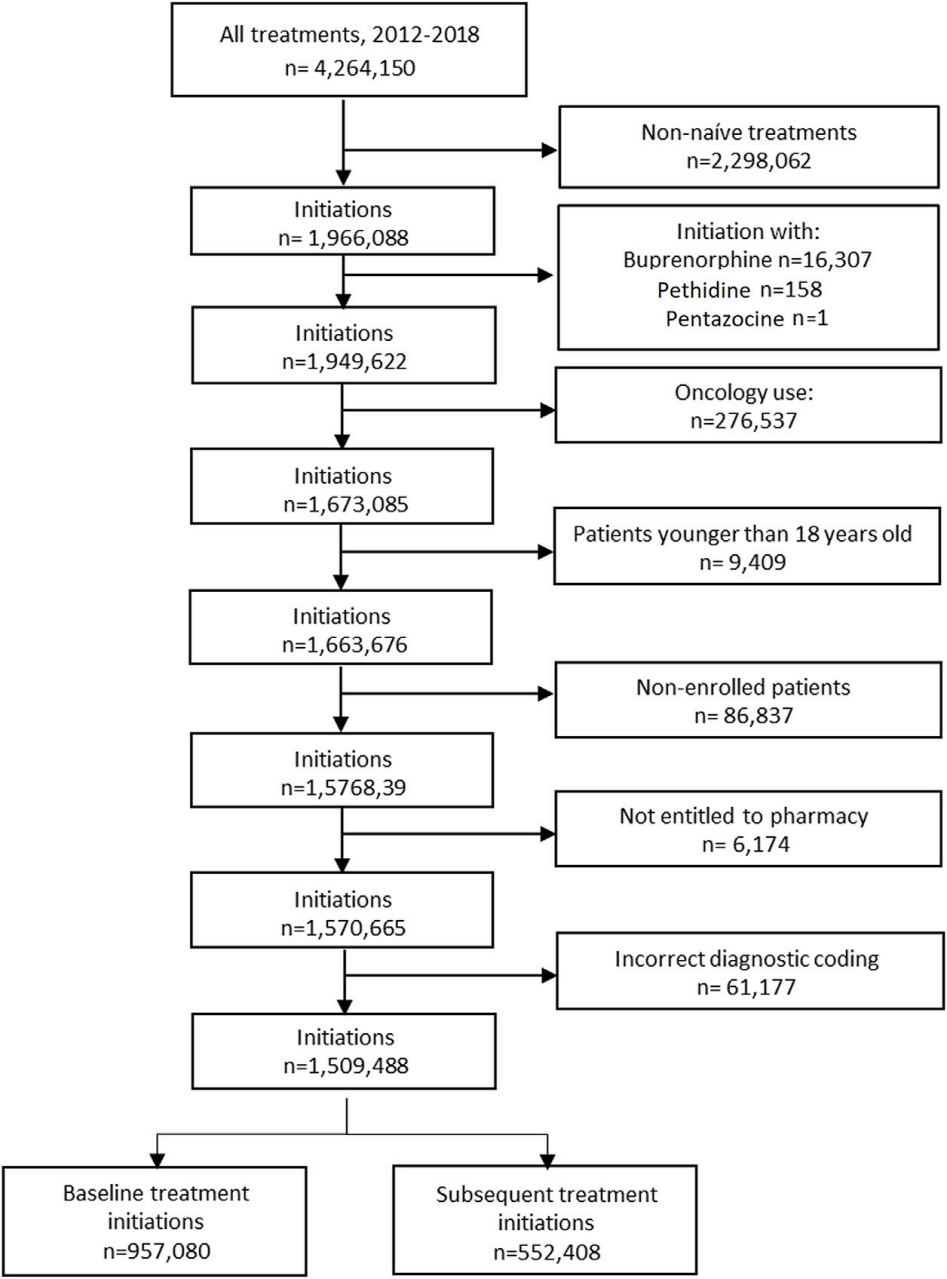
Flowchart.

The study took place in the region of Valencia (Spain) and, specifically, in the population covered by the public Valencia Health System (VHS), which covers about 97% of the region’s population. We included all adult patients aged 18 years old and over who initiated opioid treatment from 1 January 2012, to 31 December 2018. Treatment initiation (index date) was defined asthe date when an opioid treatment was prescribed, without having an opioid prescription in the previous 6 months. Accordingly, patients could initiate once (baseline initiation) or more than once (subsequent initiation) during the study period. Opioids included were classified as: long-acting (morphine, fentanyl, oxycodone, hydromorphone, tapentadol), short-acting (morphine, oxycodone), ultrafast (transmucosal fentanyl formulation), tramadol and codeine (see Supplemental Material S1). Low-dose codeine presentations containing less than 30 mg were excluded, on the rational that the maximum analgesic strength of 240 mg/day recommended by a wide range of clinical guidelines for its use as second step treatment of the World Health Organisation’s analgesic ladder cannot be reached with these. Other opioids excluded from analysis were methadone (which use is restricted to drug addiction programs in Spain), buprenorphine (to avoid confounding with addiction indications and to focus on non-cancer pain indications), pethidine and pentazocine due to marginal volume. Patients with treatment of cancer indication or with active cancer diagnostic codes in the electronic medical record of the region were also excluded. Finally, people without VHS healthcare coverage (mainly certain Spanish government employees whose prescriptions are reimbursed by mutual societies for civil servants and are thus not included in the pharmacy databases of the VHS), and patients not registered in the municipal census (non-residents or temporary residents), were excluded because of limitations on follow-up (see Figure 1). Both specialists and primary care physicians can prescribe opioids in Spain, and dispensation is subject to a tight, formal control and registry. Finally, clinical guidance in Spain is aligned with general, internationally shared recommendations on the use of prescription opioids for pain management, such as the adherence to the World Health Organisation analgesic ladder and the endorsement of the use of opioids only after employing non-opioid alternatives, the selection of short-acting morphine as third step treatment of choice, or the recommendation of establishing sort-term treatments and to avoid chronic opioid prescription.

### Data sources

Data were obtained from the VHS Integrated Databases (VID). The VID is the result of the linkage, by means of a single personal identification number, of a set of publicly owned, population-based healthcare, clinical and administrative electronic databases in Valencia, which has provided comprehensive information for the region’s five million inhabitants since 2008 (García-Sempere et al., 2020). The VID includes sociodemographic and administrative data (sex, age, nationality) as well as healthcare information such as diagnoses, procedures, laboratory data, pharmaceutical prescriptions and dispensing (including brand and generic name, formulation, strength, and dosing schedule/regimen), hospitalizations, mortality, healthcare utilization and public health data. The VID also includes a set of specific associated databases or registries with population-wide information on significant care areas such as cancer, rare disease, vaccines and imaging data (García-Sempere et al., 2020).

### Variables

We included patient socio demographic and lifestyle variables (age, sex, nationality, income range, use of alcohol and tobacco), comorbidities active at baseline (hypertension, ischemic cardiomyopathy, heart failure, COPD, diabetes, renal and liver disease, dementia and depression), and presence of surgical procedure in the 30 days before the index date.

Treatment initiation was characterised using the following variables: choice of drug, duration, number of prescriptions (treatments for one specific medication in VID may be established for as long as 12 months and may be extended for longer periods, but prescriptions are commonly refilled on a monthly basis or shorter), MME, daily MME indication of use, previous use of NSAIDS and non-opioid analgesics (defined as having received at least one prescription for NSAIDS or analgesics in the 30 days before the index date), and concomitant use (or overlap) of benzodiazepines, gabapentinoids or antipsychotics (overlap was ascertained when patients received at least one prescription for these drugs with days’ supply overlapping with the index date or initiation date of opioid use; see Supplemental Material S1 for International Classification of Diseases (ICD) and Anatomical Therapeutic Chemical (ATC) codes used for covariates and drugs included and Supplemental Material S2 for detail on the identification of surgical procedure). To estimate MME and daily MME, we used prescription information to identify milligrams prescribed and days’ supply, and an MME conversion table (see Supplemental Material S4). Indication of use was classified as: musculoskeletal (MSK) pain (back pain, osteoarthrosis, arthritis, other MSK), gastrointestinal disorders, respiratory indications (including diagnoses of flu and fever), and miscellaneous. Miscellaneous group includes several categories of pain diagnoses that individually account for a reduced percentage of patients (see Supplemental Material S3 for detail on ICD codes included in each indication category).

### Analysis

First, we described patient characteristics at baseline, overall and per opioid category of baseline initiation. Second, we described the characteristics of baseline and subsequent treatment initiation. Third, we stratified the baseline analyses for each specific drug. Fourth, we used multinominal logistic regression models to identify individual factors associated with initiating with different opioids. Tramadol was selected as reference group based on its relative volume. Variables included were age (reference group: 18–44), sex (ref. group: men), nationality (ref. group: Spanish), income range (ref. group <18.000), use of alcohol and tobacco, comorbidities and presence of surgical procedure in the 30 days before the index date (introduced as categorical variables).

### Ethics

The study was reviewed and approved by the Ethics Committee for Drug Research of the “Hospital Clínico-Universitario de Valencia” (Reference: F-CE-GeVA 14 v1.2; 2019, March 21), who waived the informed consent requirement given the retrospective design with pseudonymised data.

## Results

After applying inclusion and exclusion criteria, we ended up with 957,080 patients initiating 1,509,488 opioid treatments, of which 552,408 were subsequent initiations (see Figure 1). For baseline initiations, 738,749 were treated with tramadol (77.19%), 157,098 with codeine (16.41%) and 58,436 (6.11%) with long-acting opioids. Mean age at baseline was 56.40 years old, ranging from 49.62 for codeine to 79.55 for short-acting users. Most patients were women (58.59%), and 75.90% earned less than 18.000 euros/year; 39.72% had hypertension, 15.30% had diabetes and 15.76% had depression. Among short-acting initiators, 46.44% had dementia (see Table 1).

**TABLE 1:** Patient characteristics at baseline, overall and per type of opioid, n (%).

	Total		Ultrafast		Short-acting		Long-acting		Tramadol		Codeine		*p*-Value
	N = 957,080		N = 1,279 (0.13%)		N = 1,518 (0.16%)		N = 58,436 (6.11%)		N = 738,749 (77.19%)		N = 157,098 (16.41%)		
Mean age (SD)	56.40 (17.59)	(56.37, 56.44)	64.39 (20.09)	(63.29, 65.49)	79.55 (17.41)	(78.68, 80.43)	65.63 (16.72)	(65.49, 65.76)	57.06 (17.19)	(57.02, 57.09)	49.62 (17.31)	(49.53, 49.70)	<0.001
Age range													
*18–44*	274,017	(28.63%)	258	(20.17%)	99	(6.52%)	7,850	(13.43%)	198,115	(26.82%)	67,695	(43.09%)	<0.001
*45–64*	351,979	(36.78%)	355	(27.76%)	164	(10.80%)	18,379	(31.45%)	278,324	(37.68%)	54,757	(34.86%)	
*65–74*	167,985	(17.55%)	193	(15.09%)	126	(8.30%)	12,236	(20.94%)	133,705	(18.10%)	21,725	(13.83%)	
*≥75*	163,099	(17.04%)	473	(36.98%)	1,129	(74.37%)	19,971	(34.18%)	128,605	(17.41%)	12,921	(8.22%)	
Sex (men)	396,299	(41.41%)	620	(48.48%)	623	(41.04%)	21,868	(37.42%)	308,223	(41.72%)	64,965	(41.35%)	<0.001
Nationality													
*Spain*	802,751	(83.88%)	1,138	(88.98%)	1,388	(91.44%)	50,896	(87.10%)	615,821	(83.36%)	133,508	(84.98%)	<0.001
*European*	68,662	(7.17%)	62	(4.85%)	56	(3.69%)	4,060	(6.95%)	55,495	(7.51%)	8,989	(5.72%)	
*Non-European*	71,233	(7.44%)	57	(4.46%)	28	(1.84%)	2,513	(4.30%)	56,002	(7.58%)	12,633	(8.04%)	
*Unknown*	14,434	(1.51%)	22	(1.72%)	46	(3.03%)	967	(1.65%)	11,431	(1.55%)	1,968	(1.25%)	
Income level													
*<18.000*	726,469	(75.90%)	955	(74.67%)	1,202	(79.18%)	44,638	(76.39%)	560,118	(75.82%)	119,556	(76.10%)	<0.001
*18.000–100.000*	162,101	(16.94%)	234	(18.30%)	209	(13.77%)	9,609	(16.44%)	125,217	(16.95%)	26,832	(17.08%)	
*>100.000*	1,796	(0.19%)	5	(0.39%)	9	(0.59%)	127	(0.22%)	1,425	(0.19%)	230	(0.15%)	
*No resources*	62,663	(6.55%)	70	(5.47%)	63	(4.15%)	3,658	(6.26%)	48,745	(6.60%)	10,127	(6.45%)	
*Unknown*	4,051	(0.42%)	15	(1.17%)	35	(2.31%)	404	(0.69%)	3,244	(0.44%)	353	(0.22%)	
Comorbidities													
*Surgical procedure*	18,696	(1.95%)	79	(6.18%)	184	(12.12%)	2,038	(3.49%)	15,718	(2.13%)	677	(0.43%)	<0.001
*Hypertension*	380,106	(39.72%)	627	(49.02%)	985	(64.89%)	31,757	(54.34%)	302,091	(40.89%)	44,646	(28.42%)	<0.001
*Ischemic cardiomyopathy*	49,951	(5.22%)	125	(9.77%)	231	(15.22%)	5,084	(8.70%)	39,776	(5.38%)	4,735	(3.01%)	<0.001
*Heart Failure*	28,262	(2.95%)	183	(14.31%)	392	(25.82%)	4,306	(7.37%)	21,378	(2.89%)	2,003	(1.28%)	<0.001
*COPD*	55,676	(5.82%)	163	(12.74%)	300	(19.76%)	5,896	(10.09%)	42,947	(5.81%)	6,370	(4.05%)	<0.001
*Diabetes*	146,472	(15.30%)	310	(24.24%)	446	(29.38%)	13,185	(22.56%)	115,589	(15.65%)	16,942	(10.78%)	<0.001
*Renal disease*	50,548	(5.28%)	179	(14.00%)	348	(22.92%)	5,503	(9.42%)	39,959	(5.41%)	4,559	(2.90%)	<0.001
*Liver disease*	71,943	(7.52%)	93	(7.27%)	102	(6.72%)	4,876	(8.34%)	56,710	(7.68%)	10,162	(6.47%)	<0.001
*Dementia*	28,071	(2.93%)	176	(13.76%)	705	(46.44%)	4,608	(7.89%)	20,605	(2.79%)	1,977	(1.26%)	<0.001
*Depression*	150,806	(15.76%)	223	(17.44%)	315	(20.75%)	12,375	(21.18%)	117.23	(15.87%)	20,663	(13.15%)	<0.001
Lifestyle													
*Alcohol use*	21.54	(2.25%)	44	(3.44%)	44	(2.90%)	1,462	(2.50%)	17,258	(2.34%)	2,732	(1.74%)	<0.001
*Tobacco use*	114,129	(11.92%)	152	(11.88%)	140	(9.22%)	6,435	(11.01%)	89,833	(12.16%)	17,569	(11.18%)	<0.001

SD, standard deviation; COPD, chronic pulmonary obstructive disease.

*p* values were estimated using Chi-square for categorical variables and Anova for continuous variables.

With regard to characteristics of baseline treatment initiation, overall median duration was 9 days, 41.82% treatments lasted less than 7 days and 11.89% lasted more than 30 days. Mean number of prescriptions was 2.35, and 25.24% of ultrafast fentanyl initiators and 23.80% of long-acting initiators received three or more prescriptions (*p* < 0.001, see Table 2). Overall, daily MME was 15.00, ranging from 40.96 for ultrafast-acting fentanyl to 13.39 for tramadol (*p* < 0.001). Codeine and tramadol initiators were vastly prescribed less than 50 daily MME (95.49% and 94.64%, respectively); 19.55% of initiators with ultrafast fentanyl received more than 120 daily MME, and 16.12% of patients initiating with long-acting and 13.57% of those starting with short-acting opioids were prescribed more than 90 daily MME (*p* < 0.001).

**TABLE 2:** Characteristics of baseline initiations, overall and per type of opioid, n (%).

	Total		Ultrafast		Short-acting		Long-acting		Tramadol		Codeine		*p*-Value
	N = 957,080		N = 1,279 (0.13%)		N = 1,518 (0.16%)		N = 58,436 (6.11%)		N = 738,749 (77.19%)		N = 157,098 (16.41%)		
Duration, median (IQR)	9.00	(5.00, 19.00)	9.00	(3.00, 29.00)	8.00	(4.00, 14.00)	29.00	(14.00, 59.00)	9.00	(5.00, 19.00)	5.00	(4.00, 6.00)	<0.001
Duration strata													
*1-3 days*	67,353	(7.04%)	330	(25.80%)	305	(20.09%)	1,382	(2.36%)	39,573	(5.36%)	25,763	(16.40%)	<0.001
*4–7 days*	332,939	(34.79%)	218	(17.04%)	394	(25.96%)	4,204	(7.19%)	227,903	(30.85%)	100,220	(63.79%)	<0.001
*8-14 days*	270,503	(28.26%)	274	(21.42%)	461	(30.37%)	11,399	(19.51%)	235,628	(31.90%)	22,741	(14.48%)	<0.001
*15–30 days*	172,494	(18.02%)	262	(20.48%)	198	(13.04%)	22,112	(37.84%)	143,822	(19.47%)	6,100	(3.88%)	<0.001
*>30 days*	113,791	(11.89%)	195	(15.25%)	160	(10.54%)	19,339	(33.09%)	91,823	(12.43%)	2,274	(1.45%)	<0.001
Number of prescriptions, mean (SD)	2.35	(5.66)	3.03	(6.64)	2.74	(6.36)	2.78	(5.62)	2.53	(6.04)	1.32	(3.04)	<0.001
Number of prescriptions, range													
*1*	628,626	(65.68%)	758	(59.27%)	891	(58.70%)	34,861	(59.66%)	454,708	(61.55%)	137,408	(87.47%)	<0.001
*2*	160,009	(16.72%)	198	(15.48%)	287	(18.91%)	9,669	(16.55%)	136,028	(18.41%)	13,827	(8.80%)	
*3 or more*	168,445	(17.60%)	323	(25.25%)	340	(22.40%)	13,906	(23.80%)	148,013	(20.04%)	5,863	(3.73%)	
MME, median (IQR)	135.00	(75.00, 225.00)	390.00	(130.00, 1024.00)	200.00	(100.00, 400.00)	1200.00	(600.00, 1800.00)	150.00	(75.00, 225.00)	135.00	(90.00, 135.00)	<0.001
Daily MME													
*Median (IQR)*	15.00	(8.33, 22.50)	40.96	(18.29, 86.45)	25.00	(12.50, 50.00)	40.68	(20.69, 64.67)	13.39	(8.33, 18.75)	22.50	(18.00, 30.00)	<0.001
*Mean (SD)*	22.85	(55.67)	105.59	(411.87)	46.40	(75.66)	70.10	(187.76)	18.10	(25.24)	26.72	(18.04)	<0.001
Daily MME, range													
*<50*	894,209	(93.43%)	696	(54.42%)	1,109	(73.06%)	38,292	(65.53%)	705,429	(95.49%)	148,683	(94.64%)	<0.001
*50–89*	41,181	(4.30%)	276	(21.58%)	203	(13.37%)	10,723	(18.35%)	25,100	(3.40%)	4,879	(3.11%)	
*90–119*	8,204	(0.86%)	57	(4.46%)	111	(7.31%)	2,339	(4.00%)	3,501	(0.47%)	2,196	(1.40%)	
*≥120*	13,486	(1.41%)	250	(19.55%)	95	(6.26%)	7,082	(12.12%)	4,719	(0.64%)	1,340	(0.85%)	
Indication of use													
*Respiratory*	125,163	(13.08%)	81	(6.33%)	299	(19.70%)	1,615	(2.76%)	23,295	(3.15%)	99,873	(63.57%)	<0.001
*Gastrointestinal pain*	43,002	(4.49%)	110	(8.60%)	45	(2.96%)	1,364	(2.33%)	37,636	(5.09%)	3,847	(2.45%)	<0.001
*Osteoarticular*	172,673	(18.04%)	129	(10.09%)	43	(2.83%)	13,235	(22.65%)	152,243	(20.61%)	7,023	(4.47%)	<0.001
*Back pain*	265.58	(27.75%)	137	(10.71%)	38	(2.50%)	18,751	(32.09%)	234,824	(31.79%)	11.83	(7.53%)	<0.001
*Rheumatoid pain*	70,459	(7.36%)	40	(3.13%)	21	(1.38%)	3,618	(6.19%)	63,341	(8.57%)	3,439	(2.19%)	<0.001
*Other MSK*	113,899	(11.90%)	196	(15.32%)	74	(4.87%)	5,398	(9.24%)	100,486	(13.60%)	7,745	(4.93%)	<0.001
*Miscellaneous*	166,304	(17.38%)	586	(45.82%)	998	(65.74%)	14,455	(24.74%)	126,924	(17.18%)	23,341	(14.86%)	<0.001
Previous use of medication													
*NSAIDs or analgesics*	464,174	(48.50%)	681	(53.24%)	615	(40.51%)	32,668	(55.90%)	393,752	(53.30%)	36,458	(23.21%)	<0.001
Overlapping medication													
*Benzodiazepines*	236.69	(24.73%)	366	(28.62%)	544	(35.84%)	19,958	(34.15%)	190,197	(25.75%)	25,625	(16.31%)	<0.001
*Gabapentinoids*	44,052	(4.60%)	80	(6.25%)	46	(3.03%)	6,449	(11.04%)	35,252	(4.77%)	2,225	(1.42%)	<0.001
*Antipsychotics*	22,983	(2.40%)	132	(10.32%)	431	(28.39%)	3,029	(5.18%)	16,709	(2.26%)	2,682	(1.71%)	<0.001

IQR: interquartile range, SD: standard deviation; MME: morphine milligram equivalent; NSAID: Non-steroidal anti-inflammatory drugs, MSK: musculoskeletal.

*p* values were estimated using Chi-square for categorical variables and Anova for continuous variables.

MSK indications accounted for 65.05% of opioid use. Within MSK pain, back pain was the most prevalent (42.66% of MSK diagnoses), accounting for almost a third of the use of long-acting opioids (32.09% of patients) and tramadol (31.79%, *p* < 0.001). For codeine, 63.57% of use was for respiratory disorders. Short-acting (65.74%) and ultra-fast (45.82%) opioids were used in a wide miscellaneous group of indications (see Supplemental Material S3). Half of patients (48.50%) received NSAIDS or analgesics in the month before starting opioids. Overlapped use with benzodiazepines was observed in 24.73% of baseline initiations (35.84%, 34.15%, and 25.75% of patients initiating short-acting opioids, long-acting opioids, and tramadol, respectively). Overlap with gabapentinoids was present in 11.04% of initiations with long-acting opioids, and 28.39% of short-acting initiators used antipsychotics concomitantly (see Table 2).

In analyses for specific drugs, tapentadol was the most prescribed long-acting opioid in the period (28,787 or 49.26% of initiations with long-acting drugs), followed by oxycodone (26.72%) and fentanyl (21.75%, see Supplemental Material S6). In subsequent initiations, 55.48% of treatments included three or more prescriptions (vs. 17.60% in baseline initiations) and risk of overlap was increased for benzodiazepines (32.36% in subsequent initiation vs. 24.73 in baseline initiation, *p* < 001) and gabapentinoids (subsequent: 6.31% vs. baseline 4.60%, *p* < 0.001 (see Supplemental Material S7).

**FIGURE 2:**
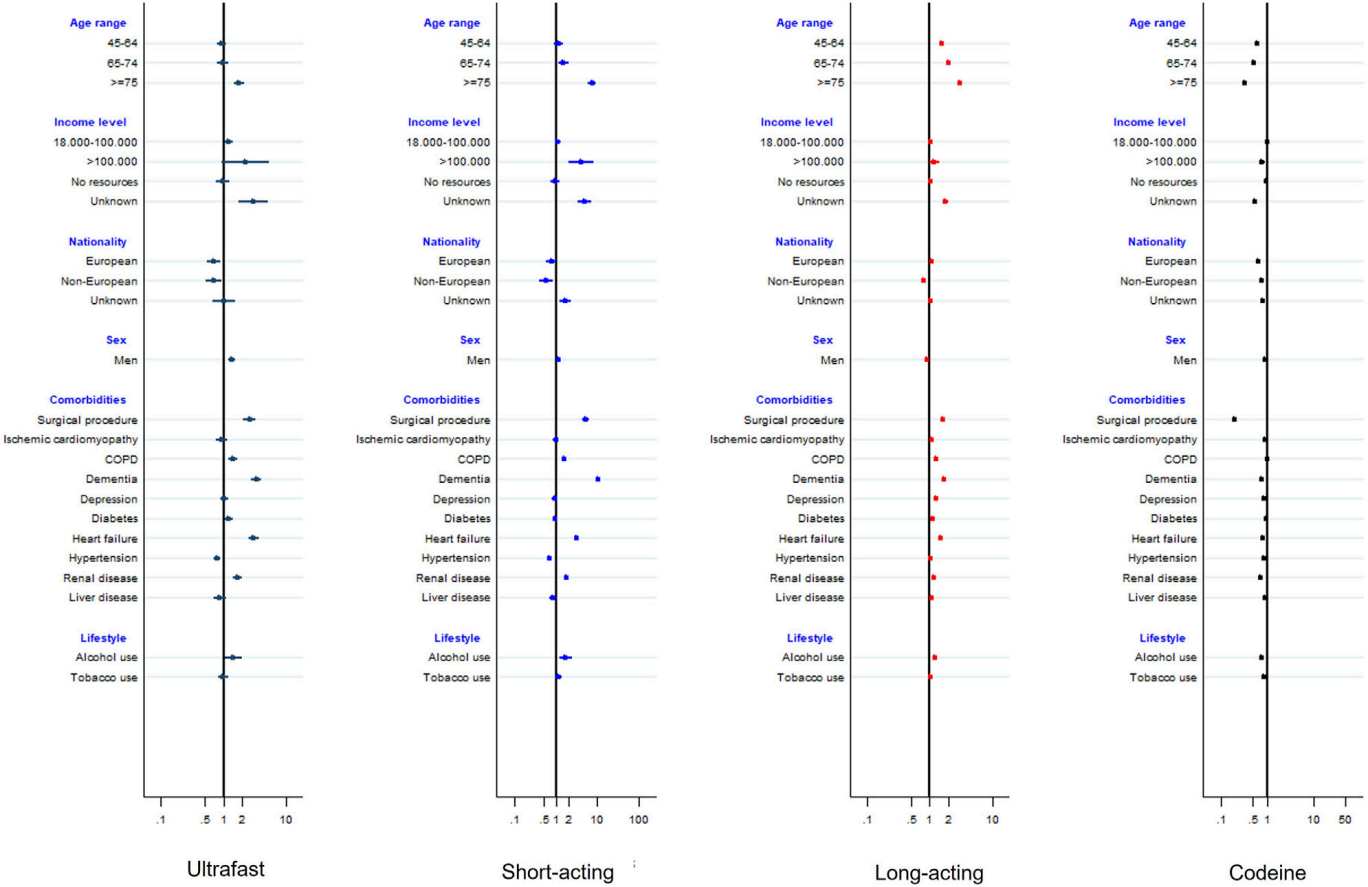
Forest plot of the multinomial regression (reference: tramadol).

In multinomial regression models, when compared to tramadol users, patients initiating with strong opioids were more likely to be 75 years old and over (RRR: 7.34 for short-acting, 2.88 for long-acting and 1.77 for ultrafast acting initiators, *p* < 0.001), whereas patients starting with codeine were younger (RRR: 0.33, *p* < 0.001). Patients initiating with long-acting, short-acting and ultrafast opioids were more likely to have had previous surgery (RRR: 1.61, 5.13 and 2.60, respectively), COPD (RRR: 1.24.1.60 and 1.42, respectively, *p* < 0.001), dementia (RRR: 1.68, 10.33 and 3.33, respectively, *p* < 0.001), congestive heart failure (RRR: 1.47, 3.13 and 3.04, respectively, *p* < 0.001), kidney disease (RRR: 1.16, 1.75 and 1.65, respectively, *p* < 0.001) and to have a record of alcohol consumption (RRR: 1.19, 1.73 and 1.44, respectively, *p* = 0.024 for ultrafast and *p* < 0.001 for the others). Patients initiating with codeine were less prone to have comorbidities (*p* < 0.001 for all comorbidities studied), except for COPD (RRR: 1.04, *p* = 0.007), and used less tobacco (RRR: 0.85, *p* < 0.001) and alcohol (RRR: 0.77, *p* < 0.001, see Figure 2 and Supplementary Material S4).

## Discussion

This study, involving almost a million of distinct adult patients that initiated more than 1.5 million opioid treatments over a 7-year period, showed that initiation with tramadol accounted for more than three-quarters of cases, followed by codeine, and long-term strong opioids (the last accounting for 6.11% and 9.34% of baseline and subsequent initiations), and that initiation with short-acting and ultrafast opioids was marginal. Tramadol and codeine are step 2 drugs for pain control; therefore, it is expected that they account for most opioid therapy initiations. However, only a half of patients initiating with tramadol received previous NSAIDs or analgesics, which would constitute appropriate care in a vast number of cases. Only one-fifth of codeine patients had previous NSAID/analgesic, but this may be explained by a different pattern of use. Codeine, used in combination with paracetamol, appears to be prescribed generally to healthier patients for milder conditions (mostly respiratory indications such as cough, fever or flu) and previous NSAIDS may not be indicated. Even so, this pattern of use of a step 2 opioid for mild conditions should be reviewed. Dosage in terms of MME in initiations with tramadol was slightly higher than with codeine, but tramadol initiations were longer, resulting in a daily MME for codeine being higher than for tramadol. In fact, one in five baseline initiations with tramadol, as well as with strong opioids, included three prescriptions or more, suggesting an intention to treat with opioids chronically. Strikingly, one in four baseline initiations with ultrafast fentanyl (which use in non-cancer pain is unrecommended by every clinical guideline in Spain) involved three or more prescriptions. Of course, long-acting opioids use in longer periods of time may be justified; but in this case, what may cause concern is the fact that patients seem to be initiating pain control treatment with step 3 drugs, a practice that merits attention. Also, a high percentage of patients initiating with third step opioids (long-acting,-short-acting, and ultrafast opioids) received more than 90 daily MME, which could be considered as high-risk prescription. Interestingly, subsequent initiations were generally longer, and risk of hazardous overlapping with other therapies was increased, showing both a trend of intensification of therapy and of potential inappropriate prescription over time.

Short-acting morphine, recommended by clinical guidelines in our setting and abroad as first-line opioid step 3 treatment, was only used marginally and in a very specific population of very elderly patient with multiple chronic conditions. Short-acting oxycodone use was residual and mainly used in surgical patients (see Supplemental Material S5). The minimal use of short-acting opioids in real clinical practice and the duration of treatments, especially in the case of long-acting opioids and tramadol, suggest an intention of long-term opioid prescribing. In this way, there is a compelling distance among clinical guidelines recommendations, which support the use of short-acting morphine and prevent from using opioids chronically for non-cancer pain (Spanish Observatory, 1144; Sicras-Mainar et al., 2021; Rejas-Gutierrez et al., 2021; Fonseca et al., 2017; García-Sempere et al., 2020; SEMFyC and FAECAP, 2017; Junta de Andalucia, 2012; Junta de Andalucia, 2018; INFAC, 2022; Dowell et al., 2016; Carville et al., 2021), and real-world use in our setting. Finally, only a small fraction of patients had a surgical procedure in the 30 days before baseline initiation (although some underestimation may exist). Whilst opioid use for post-operative pain management has proved to be effective in reducing short-term pain after surgery (Chou et al., 2016), other types of therapies are preferred for non-cancer pain. Nevertheless, the volume of use of opioids in non-cancer pain may not entirely be explained by potentially inappropriate use, and could be partly justified by the unparalleled pain relief opioids may bring for certain indications and types of pain.

Short-acting opioids were prescribed to the very elderly, patients initiating with ultrafast and long-acting opioids were old and had several comorbidities, whereas codeine patients were healthier and younger, with tramadol being halfway, which can be explained by the high volume of use of tramadol in a diverse range of patients, indications and pain intensities. With regard to their therapeutic use, tramadol and long-acting opioids were used in a very similar way and mainly for MSK pain. Ultrafast and short-acting opioids were initiated in a wide array of pain conditions categorised as “miscellaneous”. In fact, this category also represents a significant number of initiations for the rest of opioids; this confirms that use of opioids in non-cancer pain is widespread in several indications, which could suppose a difficulty for the implementation of strategies of rational use.

Many studies have addressed the use of prescription opioids in Europe and have raised concerns about increasing trends and concerning patterns of use. However, there is a great heterogeneity between studies in the operational definitions and the nature of data employed, and there is a lack of granular, high quality epidemiological data on opioid use that is required to implement policy responses at national or regional level and to make accurate comparisons (Kalkman et al., 2022). Also, contextual information is important (such as, for instance, indication of use) to interpret the use of a drug in a specific setting, and comparison between settings without that information should be made with caution. Despite this and even if the overall volume of opioid use has seen a stabilisation in many countries since the late 2010s, some matching trends can be ascertained across different settings in the last 20 years, such as the growing use of opioids for non-cancer indications, the high-volume use of tramadol, or the periods of high growth of tapentadol, fentanyl or oxycodone use in the European population (Morgan et al., 2008; van Amsterdam and van den Brink, 2015; Verhamme Bohnen, 2019; Alho et al., 2020; Jivraj et al., 2020; Karmali et al., 2020; Seyler et al., 2021; van Amsterdam et al., 2021). However, comparative research into the factors driving opioid use and misuse and related harm in Europe is lacking. For instance, most studies specifically describing the patterns of opioid initiation in non-cancer pain come from the US (Dobscha et al., 2013; Kern et al., 2015; Deyo et al., 2017; Shah et al., 2017; Mundkur et al., 2018; Larach et al., 2020; Hadlandsmyth et al., 2022; Weiner et al., 2022), some from other countries (Zin et al., 2019; Jani et al., 2020; Gomes et al., 2021; Pilarinos et al., 2022) and virtually none from Europe. In this sense, our study provides very much needed information about specific patterns of initiation with different opioid agents for a vast number of non-cancer indications, with rich individual and contextual information, and depicts different profiles of patients exposed to opioids. This may contribute to set priority of action and to orientate policy, and is the foundation to address reliably the study of opioid-related harm in our setting and in Europe, such as the long-term use, abuse, dependence, and other potential adverse outcomes associated to opioid initiation.

Our study is subject to some limitations. First, the VID databases gather several real-world clinical practice data and contain information as registered by health professionals during routine clinical practice, but data are not specifically prepared for research. In this sense, studies based on real-world clinical information like the VID are at risk of well-known biases such a differential recording, misclassification bias or missing data. However, the data contained in the databases in VID that have been essential to this work, which are the Information Population System (the official registry of the regions’ population healthcare coverage) and the outpatient (primary care and specialist) pharmaceutical prescription information (which is used for billing purposes and includes paperless electronic prescription, the registration of any dispensation in any community pharmacy, and reimbursement to pharmacies in a traceable way for each pharmaceutical package and each patient) are of a very high quality, reliability and completeness. . Second, by design we excluded some groups of patients, such as initiators with excluded opioids or those who initiated two opioids at the same time. Also, we failed to include 4% of initiations in the analyses due to incorrect prescription diagnostic coding, which hindered the classification of indication of use of opioid initiations. In this sense, we are missing a fraction of the population treated. Third, some of the categories we used to define indication of use may be hard to interpret, as they include a great number of diverse indications and of ICD codes employed to initiate opioids. Also, coding decision-making in real world practice may be subject to variability, a potential bias frequent in retrospective real-world studies. Fourth, information on inpatient medication is not available in VID, which may result in some misestimation. Fifth, our results are merely descriptive, and further investigation on the association of patterns of initiation with subsequent episodes of care and outcomes is warranted. Finally, the generalization of our results to other settings outside Spain, or even to other Spanish regions, should be approached with great caution, as, again, contextual factors may play an important role in initiation patterns.

In conclusion, we showed that opioids are initiated for a vast array of non-oncological indications and that, despite clinical guidelines, short-acting opioids are used marginally, and a significant number of patients is exposed to potentially high-risk patterns of initiation, such as treatments lasting more than 14 days, treatments surpassing 50 daily MMEs, initiating with long-acting opioids, or hazardous overlapping with other therapies. These findings point at some potential areas for intervention to improve the appropriateness of opioid use in noncancer pain. Also, the use of tramadol should be monitored according to its high volume of use, and the potentially avoidable use of codeine in mild pain or non-pain indications, or the initiation of pain therapy with opioids instead of non-opioid agents, addressed. Whether these observed patterns may put patients at risk of chronic use of opioids and opioid-related harm warrants further investigation.

## Data Availability

The datasets presented in this article are not readily available because The datasets presented in this article are not readily available because legal restrictions on sharing the data set apply as regulated by the Valencia regional government by means of legal resolution by the Valencia Health Agency [2009/13312] which forbids the dissemination of data to third parties (accessible at: http://www.san.gva.es/documents/152919/157920/resolucionsolicituddatos.pdf). Upon request, authors can allow access to the databases in order to verify the accuracy of the analysis or the reproducibility of the study. Requests to access the datasets should be directed to Management Office of the Data Commission in the Valencia Health Agency (email: solicitud_datos@gva.es; telephone numbers: +34 961-928207; +34 961-928198).
