# Supplementary material for: Real-world patterns of opioid therapy initiation in Spain, 2012–2018: A population-based, retrospective cohort study with 957,080 patients and 1,509,488 initiations
Source: Front Pharmacol. 2022 Nov 16;13:1025340. doi: 10.3389/fphar.2022.1025340 (PMC9709437; doi:10.3389/fphar.2022.1025340)
Supplement: Supplementary file 1 [file Table1.docx]

**Real-world patterns of opioid therapy initiation in Spain, 2012-2018: a population-based, retrospective cohort study with 957,080 patients and 1,509,488 initiations.**

Supplemental Material

Table S1. ICD and ATC codes used for covariates and drugs included in the study.

Table S2. Identification of surgical procedures.

Table S3. Indications of use – ICD codes.

Table S4. MME conversion table

Table S4. Results of the multinomial regression model.

Table S5. Patient characteristics at baseline for specific short-acting and long-acting drugs.

Table S6. Characteristics of baseline initiations for specific short-acting and long-acting drugs.

Table S7. Characteristics of subsequent initiation, overall and per type of opioid.

**Table S1. ICD and ATC codes used for covariates and drugs included in the study.**

| **ATC codes used for medication** | | |
| --- | --- | --- |
| Opioids | N02A, except N02AE01 (buprenorphine), N02AB02 (petidine), N02AX02 (pentazocine) | |
| Benzodiazepines | N05BA, N05CD, N05CF | |
| Gabapentinoids | N03AX | |
| Antipsychotics | N05A | |
| Antiinflammatory and antirheumatic products, non-steroids | M01A | |
| Other analgesics and antipyretics | N02B | |
| **ICD codes used for comorbidities** | **International Classification of Disease, 9th edition, ICD-9-CM** | **International Classification of Disease, 10th edition, ICD-10** |
| Congestive heart failure | 428.xx, 398.91, 402.01, 402.11, 402.91, 404.01, 404.11, 404.03, 404.13, 404.91, 404.93, 425.4x | I50.x, I11.0, I13.0, I13.2, I42.0 |
| Hypertension | 401.xx- 405.xx, 437.2x | I10.x, I11.x, I12.x, I13.x, I15.x, I67.4 |
| CHD | 410.xx, 411.xx, 412.xx, 413.xx, 414.xx | I20.x, I21.x, I22.x, I23.x, I24.x, I25.x |
| CODP | 491.xx, 492.xx, 496.xx, 493.20, 493.21, 493.22 | J41.0, J41.1, J41.9, J42.x, J43.0, J43.1, J43.2, J43.8, J43.9, J44.0, J44.1, J44.8, J44.9 |
| Diabetes | 249.xx, 250.xx, 648.0 | E08.x- E11.x, E13.x |
| Renal disease | 403.xx, 404.xx, 580.xx- 589.xx, 590.1, 792.5, 753.x, V42.0, V45.1, V56.xx | I12.x, I13.x, N00.x- N05.x, N07.x, N11.x, N14.x, N17.x-N19.x,Q61.x, Z49.x, Z94.0, Z99.2 , Z91.15 |
| Liver disease | 070.0, 070.2x, 070.4x, 070.6x, 070.71, 570.xx- 573.xx 782.4, 789.1, 789.5, 790.4, 790.5, 794.8, V42.7 | B150.x,B160.x, B162.x, B190.x, K70.x- K77.x I85.x, R94.5, Z94.4 R17.x, R18.x, R74.x |
| Dementia | 290.xx, 294.xx, 330.xx, 331.xx | F00.x,F01.x, F02.x, F03.x, G30.x, G31.x |
| Depression | 296.2x, 296.3x, 298.0x, 300.4x, 301.12, 311.xx | F32.x, F33.x, F34.1 |
| Alcohol | 291.xx, 303.xx, 305.0x, 980.0, 357.5 | E52.x, F10.x, G31.2, G62.1, G72.1, I42.6, K29.2, K70.x, K86.0, O35.4, T51.x, Z71.4 |
| Tobacco | 305.1x, 989.84, V15.82 | F17.x,T65.2 |

**Table S2. Identification of surgical procedures via ICD codes.**

| **ICD9 procedures** | **ICD10 procedures** |
| --- | --- |
| 00 Procedures and interventions, not elsewhere classified | 00 Central Nervous System and Cranial Nerves |
| 01-05 Operations on the nervous system | 01 Peripheral Nervous System |
| 06-07 Operations on the endocrine system | 02 Heart and Great Vessels |
| 08-16 Operations on the eye | 03 Upper Arteries |
| 18-20 Operations on the ear | 04 Lower Arteries |
| 21-29 Operations on the nose, mouth, and pharynx | 05 Upper Veins |
| 30-34 Operations on the respiratory system | 06 Lower Veins |
| 35-39 Operations on the cardiovascular system | 07 Lymphatic and Hemic Systems |
| 40-41 Operations on the hemic and lymphatic system | 08 Eye |
| 42-54 Operations on the digestive system | 09 Ear, Nose, Sinus |
| 55-59 Operations on the urinary system | 0B Respiratory System |
| 60-64 Operations on the male genital organs | 0C Mouth and Throat |
| 65-71 Operations on the female genital organs | 0D Gastrointestinal System |
| 76-84 Operations on the musculoskeletal system | 0F Hepatobiliary System and Pancreas |
| 85-86 Operations on the integumentary system | 0G Endocrine System |
|  | 0H Skin and Breast |
|  | 0J Subcutaneous Tissue and Fascia |
|  | 0K Muscles |
|  | 0L Tendons |
|  | 0M Bursae and Ligaments |
|  | 0N Head and Facial Bones |
|  | 0P Upper Bones |
|  | 0Q Lower Bones |
|  | 0R Upper Joints |
|  | 0S Lower Joints |
|  | 0T Urinary System |
|  | 0U Female Reproductive System |
|  | 0V Male Reproductive System |
|  | 0W Anatomical Regions, General |
|  | 0X Anatomical Regions, Upper Extremities |
|  | 0Y Anatomical Regions, Lower Extremities |

*Surgical procedures were identified using the ICD 9 and ICD 10 procedure codes listed, in the 30 days before the index date of the baseline initiation. Additionally, we also included patients discharged from a hospital surgical service in that same period.*

| **Table S3. ICD9 codes used for categories of indication of use** | |
| --- | --- |
|  | |
| **Indication** | **ICD9 codes** |
| Respiratory disorders | 460* - 519* |
| GastrointestinaI disorders | 520* - 579* |
| Osteoarticular pain | 696.0, 710*, 711*, 712. *, 713.*, 714*,715.*, 716.0*, 716.1*, 716.2*, 716.4*, 716.3*, 716.5*, 716.6*, 716.8*, 716.9*, 717*, 718*, 719*, 730.0*, 730.1*, 730.2*, 730.3*, 730.7*,730.8*,730.9*,731*,732*,733*, 734*,735*,736*,737*,738*,739* |
| Back pain | 720*, 721*, 722*, 723*, 724* |
| Reumathoid / arthritis | 725, 726*, 727*,728*, 729* |
| Other musculoskeletal disorders | 756*, 784.0, 784.1, 784.92, 786.5*, 788.0, 788.20, 788.29, 789.0*, 789.6*, 789.7, 800-897. *, 920. *, 921.0, 921.1, 922.*, 923.*, 924.*, 925-949.*, 953.*, 954.8, 954.9, 955-957.*, 958.9*, 959.*, 997.41, 997.62, V13.4, V13.5*, V43.6* |
| Miscellaneous | Infectious Diseases: 003.23, 003.24, 009*, 015*, 036.82, 040.0, 040.81, 053*, , 054.10, 054.11, 054.12, 054.13, 054.19, 056.71, 060*, 061*, 066.40, 066.49, 072.0, 072.3, 074.1, 074.20, 074.21, 074.23, 079*, 088.81, 095.5, 095.7, 099.3, 101, 112.84, 117.5, 122*, 135, 136.0, 136.1, 136.5, 137.3 321.2 |
|  | Endocrine Disease: 244*, 245.0, 245.1, 249.60, 249.61, 249.70, 249.71, 250*, 251.5, 268.0, 268.1, 268.2, 272*, 277.1 |
|  | Nutrition: 266.0, 266.2 |
|  | Joint Disease: 135, 136.1, 274.0, 274.01, 274.00, 274.02, 274.03, 274.9, 275.01, 275.02, 275.03, 275.49, 277.2, 277.30, 277.31 |
|  | Immune Disorders: 279.50, 279.51, 279.52, 279.53 |
|  | Hematologic Disorders: 282.41, 282.42, 282.60, 282.61, 282.62, 282.63, 282.64, 282.68, 282.69, 286.0, 286.1, 289.1, 790* |
|  | Neurologic Disorders (including headache): 321.4, 322.0, 322.1, 322.2, 322.9, 324.0, 324.1, 324.9, 325, 332.0*, 336.0, 339.00, 339.01, 339.02, 339.03, 339.04, 339.05, 339.09, 339.10, 339.11, 339.12, 339.20, 339.21, 339.22, 339.3, 339.41, 339.42, 339.43, 339.44, 339.81, 339.82, 339.83, 339.84, 339.85,339.89, 340*, 341.0, 341.20, 341.21, 341.22, 346.00, 346.01, 346.02, 346.03, 346.1*, 346.2*, 346.3*, 346.4*, 346.5*, 346.6*, 346.7*, 346.8*, 346.9*, 349.0, 350.1, 350.2, 353*, 354*, 355*, 356.0, 356.2, 356.4, 356.8, 356.9, 357.0, 357.1, 357.2, 357.4, 357.5, 357.6, 357.7, 357.81, 357.82, 357.89, 357.9, 359.4, 359.5, 359.6, 359.7*, 359.8*, 359.9*, 430, 431*, 432*, 437.4, 437.6 |
|  | Pain (cause unspecified): 338.0, 338.11, 338.12, 338.18, 338.19, 338.21, 338.22, 338.28, 338.29, 338.3, 338.4, 780*, 999* |
|  | Eye Disorders: 360.03, 360.11, 360.12, 376.02, 376.03, 379.91 |
|  | Ear Disorders: 380*, 382*,383*, 388* |
|  | Cardiovascular Disorders: 390*, 391*, 393*, 401*, 415.1*, 420*, 422*, 429.0, 443.1, 443.8*, 443.9, 444.2*, 444.8*, 444.9, 445*,446.0, 446.3, 446.4, 446.7, 447.6, 449*, 451*, 453.0, 453.1, 453.4*, 453.82, 453.83, 453.84, 453.89, 454.0, 454.1, 454.2, 454.8, 457.0, 457.1, 457.2, 459* |
|  | Genito urethral Disorders: 588.0, 590.00, 590.01, 590.10, 590.11, 590.2,590.80, 590.81, 590.9, 591*, 592.*,595*, 596.6, 596.81, 599.0, 599.6, 599.60, 599.69, 601.2, 607.3, 608.2*, 611.0, 611.71,614.1, 614.2, 614.4, 614.5, 616.5*, 614.7, 614.8, 614.9, 616.81, 616.89, 616.9, 617.* 620.5, 625*, 629.3*, 633.*, 639.0, 664.0*, 664.1*, 664.2*, 664.3*, 664.4*, 664.6*, 664.8*, 664.9*, 665.0*, 665.1*, 665.3*, 665.4*, 665.5*, 665.8*, 665.9*, 673.*, 674.1*, 674.2*, 674.3* |
|  | Skin Disorders: 682*, 683*, 686.01, 692*, 694.4, 695.2, 695.81, 705.83, 707*, 782* |
|  | Mental Disorders: 300*, 307.80, 307.81, 307.89, 311* |

|  | Other: V01-V87, Except: V13.4, V13.5*, V43.6* |
| --- | --- |

**Table S4. Morphine Milligram Equivalent (MME) conversion table**

|  | |
| --- | --- |
| **Opioid (strength in mg except where noted)** | **MME Conversion Factor*** |
| Buprenorphine, transdermal patch (MCG/HR) | 12.6 |
| Buprenorphine, tablet or film | 30 |
| Buprenorphine, film (MCG) | 0.03 |
| Codeine | 0.15 |
| Fentanyl, buccal/SL tabet or lozenge/troche (MCG) | 0.13 |
| Fentanyl, film or oral spray (MCG) | 0.18 |
| Fentanyl, nasal spray (MCG) | 0.16 |
| Fentanyl, transdermal patch (MCG/HR) | 7.2 |
| Hydromorphone | 4 |
| Morphine | 1 |
| Oxycodone | 1.5 |
| Tapentadol | 0.4 |
| Tramadol | 0.1 |
| Source: CDC 2016.  *To be used in the formula: Strength per Unit  X (Number of Units/ Days Supply) X MME conversion factor = MME/Day. Please see Documentation for additional information on using the formula with transdermal patches.  MCG: microgram; HR: hour | |

|  | Ultrafast | | | | Short-acting | | | | Long-acting | | | | Codeine | | | |
| --- | --- | --- | --- | --- | --- | --- | --- | --- | --- | --- | --- | --- | --- | --- | --- | --- |
|  | RRR | P>\|z\| | [95% Conf.Interval] | | RRR | P>\|z\| | [95% Conf.Interval] | | RRR | P>\|z\| | [95% Conf.Interval] | | RRR | P>\|z\| | [95% Conf.Interval] | |
| Age range (ref 18-44) |  |  |  |  |  |  |  |  |  |  |  |  |  |  |  |  |
| *45-64* | .9493182 | 0.543 | .8027489 | 1.122649 | 1.142462 | 0.301 | .8877564 | 1.470245 | 1.54764 | 0.000 | 1.504782 | 1.591718 | .6020204 | 0.000 | .5939469 | .6102036 |
| *65-74* | .9650301 | 0.736 | .7844892 | 1.18712 | 1.496147 | 0.004 | 1.135134 | 1.971975 | 1.978972 | 0.000 | 1.915713 | 2.044319 | .5140901 | 0.000 | .5042176 | .524156 |
| *>=75* | 1.768932 | 0.000 | 1.459548 | 2.143897 | 7.339767 | 0.000 | 5.806815 | 9.277404 | 2.978655 | 0.000 | 2.882406 | 3.078118 | .3332199 | 0.000 | .3253142 | .3413176 |
| Income level (ref <18.000e/year) | |  |  |  |  |  |  |  |  |  |  |  |  |  |  |  |
| *18,000-100,000* | 1.21829 | 0.008 | 1.053184 | 1.409279 | 1.112946 | 0.167 | .9563083 | 1.29524 | 1.023178 | 0.056 | .9994296 | 1.04749 | 1.013124 | 0.088 | .9980607 | 1.028415 |
| *>100,000* | 2.267061 | 0.068 | .9411141 | 5.461152 | 4.047022 | 0.000 | 2.065087 | 7.93109 | 1.174289 | 0.087 | .9766748 | 1.411886 | .7954945 | 0.001 | .690792 | .9160667 |
| *No resources* | .9884939 | 0.926 | .7731455 | 1.263824 | .9716648 | 0.828 | .7492158 | 1.260161 | 1.02782 | 0.131 | .9918285 | 1.065118 | .9571602 | 0.000 | .9356713 | .9791427 |
| *Unknown* | 2.997333 | 0.000 | 1.774781 | 5.062036 | 4.899448 | 0.000 | 3.356658 | 7.151336 | 1.783867 | 0.000 | 1.60745 | 1.979645 | .5498248 | 0.000 | .491653 | .6148794 |
| Nationality (ref: Spanish) |  |  |  |  |  |  |  |  |  |  |  |  |  |  |  |  |
| *European* | .6996977 | 0.006 | .541391 | .9042945 | .7868752 | 0.087 | .5981796 | 1.035095 | 1.046961 | 0.008 | 1.012208 | 1.082907 | .6676613 | 0.000 | .6521868 | .6835031 |
| *Non-European* | .7035427 | 0.014 | .5309216 | .932289 | .5851094 | 0.007 | .3966489 | .8631137 | .7881598 | 0.000 | .7551942 | .8225644 | .7885966 | 0.000 | .7722201 | .8053205 |
| *Unknown* | 1.010674 | 0.961 | .6625556 | 1.541701 | 1.697931 | 0.001 | 1.252889 | 2.301057 | 1.018864 | 0.585 | .9528252 | 1.089481 | .8164715 | 0.000 | .777534 | .8573589 |
| Sex (ref: men) | 1.348129 | 0.000 | 1.199177 | 1.515583 | 1.169918 | 0.007 | 1.044613 | 1.310254 | .906318 | 0.000 | .8897057 | .9232405 | .9300561 | 0.000 | .9192484 | .9409909 |
| Indication of use |  |  |  |  |  |  |  |  |  |  |  |  |  |  |  |  |
| *Surgical procedure* | 2.603401 | 0.000 | 2.065224 | 3.28182 | 5.133361 | 0.000 | 4.330294 | 6.085359 | 1.607485 | 0.000 | 1.531778 | 1.686933 | .2028155 | 0.000 | .187606 | .219258 |
| *Ischemic cardiomyopathy* | .9361073 | 0.522 | .7648906 | 1.14565 | .9957202 | 0.956 | .8549798 | 1.159628 | 1.048401 | 0.004 | 1.014773 | 1.083142 | .8923252 | 0.000 | .8641718 | .9213958 |
| *CODP* | 1.419641 | 0.000 | 1.192603 | 1.6899 | 1.601954 | 0.000 | 1.38577 | 1.851863 | 1.276421 | 0.000 | 1.238076 | 1.315954 | 1.039515 | 0.007 | 1.010728 | 1.069122 |
| *Dementia* | 3.331505 | 0.000 | 2.792351 | 3.974761 | 10.33027 | 0.000 | 9.185645 | 11.61752 | 1.683099 | 0.000 | 1.62523 | 1.743029 | .7870298 | 0.000 | .7501268 | .8257483 |
| *Depression* | 1.030984 | 0.693 | .8860416 | 1.199638 | .9332305 | 0.317 | .8151653 | 1.068396 | 1.260275 | 0.000 | 1.23315 | 1.287997 | .8629636 | 0.000 | .8488824 | .8772784 |
| *Diabetes* | 1.208188 | 0.008 | 1.050525 | 1.389515 | .9679519 | 0.595 | .8583012 | 1.091611 | 1.096812 | 0.000 | 1.072837 | 1.121322 | .9616985 | 0.000 | .9440207 | .9797073 |
| *Heart Failure* | 3.042847 | 0.000 | 2.534059 | 3.65379 | 3.128242 | 0.000 | 2.737993 | 3.574113 | 1.473873 | 0.000 | 1.420953 | 1.528763 | .8045734 | 0.000 | .7668286 | .8441761 |
| *Hypertension* | .7903464 | 0.001 | .6912537 | .9036442 | .7060756 | 0.000 | .62301 | .8002163 | 1.018671 | 0.079 | .9978701 | 1.039906 | .8746484 | 0.000 | .8620189 | .8874629 |
| *Renal disease* | 1.650556 | 0.000 | 1.388214 | 1.962475 | 1.754378 | 0.000 | 1.536127 | 2.003637 | 1.16945 | 0.000 | 1.133561 | 1.206475 | .743262 | 0.000 | .7197437 | .7675487 |
| *Liver disease* | .8701301 | 0.211 | .6996602 | 1.082134 | .8741956 | 0.207 | .7094843 | 1.077146 | 1.04403 | 0.007 | 1.011862 | 1.077221 | .9360483 | 0.000 | .9153449 | .95722 |
| *Alchohol use* | 1.438517 | 0.024 | 1.049811 | 1.971146 | 1.726829 | 0.001 | 1.253068 | 2.37971 | 1.190409 | 0.000 | 1.125127 | 1.259478 | .7748962 | 0.000 | .7429847 | .8081782 |
| *Tobacco use* | .9903986 | 0.916 | .8283398 | 1.184163 | 1.157641 | 0.125 | .960104 | 1.39582 | 1.024281 | 0.098 | .9955857 | 1.053804 | .8535458 | 0.000 | .8384346 | .8689293 |
| Constant | .0010872 | 0.000 | .0009382 | .0012597 | .0004154 | 0.000 | .0003391 | .0005088 | .0394749 | 0.000 | .0384902 | .0404849 | .408358 | 0.000 | .4036143 | .4131574 |

**Table S5. Results of the multinomial regression model**

| **Table S6. Patient characteristics at baseline for specific short-acting and long-acting drugs.** | | | | | | | | | | | | | | |  |
| --- | --- | --- | --- | --- | --- | --- | --- | --- | --- | --- | --- | --- | --- | --- | --- |
|  | **Short-acting** | | | | **Long-acting** | | | | | | | | | |  |
|  | **Morphine** | | **Oxicodone** | | **Fentanyl** | | **Hydromorphone** | | **Morphine** | | **Oxicodone** | | **Tapentadol** | | **p value** |
|  | N=1,258 (82.87%) | | N=260 (17.13%) | | N=12,710 (21.75%) | | N=579 (0.99%) | | N=743 (1.27%) | | N=15,617 (26.72%) | | N=28,787 (49.26%) | |  |
| Mean age (SD) | 84.77(11.49) | (84.13,85.40) | 54.32(19.07) | (51.99,56.65) | 75.22(15.05) | (74.95,75.48) | 65.66(16.06) | (64.35,66.97) | 69.44(16.8) | (68.23,70.65) | 64.24(15.86) | (63.99,64.49) | 62.05(16.25) | (61.86,62.23) | <0.001 |
| Age range |  |  |  |  |  |  |  |  |  |  |  |  |  |  |  |
| *18-44* | 20 | (1.59%) | 79 | (30.38%) | 672 | (5.29%) | 69 | (11.92%) | 77 | (10.36%) | 2,145 | (13.74%) | 4,887 | (16.98%) | <0.001 |
| *45-64* | 66 | (5.25%) | 98 | (37.69%) | 2,092 | (16.46%) | 185 | (31.95%) | 179 | (24.09%) | 5,320 | (34.07%) | 10,603 | (36.83%) |  |
| *65-74* | 84 | (6.68%) | 42 | (16.15%) | 2,300 | (18.10%) | 132 | (22.80%) | 169 | (22.75%) | 3,489 | (22.34%) | 6,146 | (21.35%) |  |
| *>=75* | 1,088 | (86.49%) | 41 | (15.77%) | 7,646 | (60.16%) | 193 | (33.33%) | 318 | (42.80%) | 4,663 | (29.86%) | 7,151 | (24.84%) |  |
| Sex (men) | 482 | (38.31%) | 141 | (54.23%) | 4,48 | (35.25%) | 173 | (29.88%) | 408 | (54.91%) | 5,541 | (35.48%) | 11,266 | (39.14%) | 0.004 |
| Nationality |  |  |  |  |  |  |  |  |  |  |  |  |  |  |  |
| *Spain* | 1,169 | (92.93%) | 219 | (84.23%) | 11,426 | (89.90%) | 497 | (85.84%) | 614 | (82.64%) | 13,670 | (87.53%) | 24,689 | (85.76%) | <0.001 |
| *European* | 36 | (2.86%) | 20 | (7.69%) | 760 | (5.98%) | 42 | (7.25%) | 96 | (12.92%) | 1,045 | (6.69%) | 2,117 | (7.35%) |  |
| *Non-European* | 11 | (0.87%) | 17 | (6.54%) | 309 | (2.43%) | 30 | (5.18%) | 20 | (2.69%) | 663 | (4.25%) | 1,491 | (5.18%) |  |
| *Unknown* | 42 | (3.34%) | 4 | (1.54%) | 215 | (1.69%) | 10 | (1.73%) | 13 | (1.75%) | 239 | (1.53%) | 490 | (1.70%) |  |
| Income level |  |  |  |  |  |  |  |  |  |  |  |  |  |  |  |
| *<18.000* | 1,016 | (80.76%) | 186 | (71.54%) | 10,135 | (79.74%) | 443 | (76.51%) | 519 | (69.85%) | 11,956 | (76.56%) | 21,585 | (74.98%) | <0.001 |
| *18.000-100.000* | 154 | (12.24%) | 55 | (21.15%) | 1,582 | (12.45%) | 76 | (13.13%) | 142 | (19.11%) | 2,555 | (16.36%) | 5,254 | (18.25%) |  |
| *>100.000* | 7 | (0.56%) | 2 | (0.77%) | 24 | (0.19%) | 2 | (0.35%) | 1 | (0.13%) | 32 | (0.20%) | 68 | (0.24%) |  |
| *No resources* | 47 | (3.74%) | 16 | (6.15%) | 761 | (5.99%) | 48 | (8.29%) | 63 | (8.48%) | 1,018 | (6.52%) | 1,768 | (6.14%) |  |
| *Unknown* | 34 | (2.70%) | 1 | (0.38%) | 208 | (1.64%) | 10 | (1.73%) | 18 | (2.42%) | 56 | (0.36%) | 112 | (0.39%) |  |
| Comorbidities |  |  |  |  |  |  |  |  |  |  |  |  |  |  |  |
| *Surgical procedure* | 60 | (4.77%) | 124 | (47.69%) | 811 | (6.38%) | 11 | (1.90%) | 44 | (5.92%) | 435 | (2.79%) | 737 | (2.56%) | <0.001 |
| *Hypertension* | 891 | (70.83%) | 94 | (36.15%) | 8,700 | (68.45%) | 324 | (55.96%) | 418 | (56.26%) | 8,312 | (53.22%) | 14,003 | (48.64%) | <0.001 |
| *Ischemic cardiomyopathy* | 218 | (17.33%) | 13 | (5.00%) | 1,775 | (13.97%) | 44 | (7.60%) | 113 | (15.21%) | 1,186 | (7.59%) | 1,966 | (6.83%) | <0.001 |
| *Heart Failure* | 381 | (30.29%) | 11 | (4.23%) | 2,076 | (16.33%) | 37 | (6.39%) | 144 | (19.38%) | 802 | (5.14%) | 1,247 | (4.33%) | <0.001 |
| *COPD* | 253 | (20.11%) | 47 | (18.08%) | 2,002 | (15.75%) | 60 | (10.36%) | 222 | (29.88%) | 1,379 | (8.83%) | 2,233 | (7.76%) | <0.001 |
| *Diabetes* | 409 | (32.51%) | 37 | (14.23%) | 3,978 | (31.30%) | 132 | (22.80%) | 191 | (25.71%) | 3,340 | (21.39%) | 5,544 | (19.26%) | <0.001 |
| *Renal disease* | 333 | (26.47%) | 15 | (5.77%) | 2,237 | (17.60%) | 29 | (5.01%) | 120 | (16.15%) | 1,081 | (6.92%) | 2,036 | (7.07%) | <0.001 |
| *Liver disease* | 79 | (6.28%) | 23 | (8.85%) | 1,127 | (8.87%) | 38 | (6.56%) | 72 | (9.69%) | 1,218 | (7.80%) | 2,421 | (8.41%) | 0.027 |
| *Dementia* | 694 | (55.17%) | 11 | (4.23%) | 2,464 | (19.39%) | 32 | (5.53%) | 86 | (11.57%) | 794 | (5.08%) | 1,232 | (4.28%) | <0.001 |
| *Depression* | 277 | (22.02%) | 38 | (14.62%) | 2,838 | (22.33%) | 137 | (23.66%) | 158 | (21.27%) | 3,491 | (22.35%) | 5,751 | (19.98%) | 0.712 |
| Lifestyle |  |  |  |  |  |  |  |  |  |  |  |  |  |  |  |
| *Alcohol use* | 29 | (2.31%) | 15 | (5.77%) | 412 | (3.24%) | 13 | (2.25%) | 34 | (4.58%) | 290 | (1.86%) | 713 | (2.48%) | 0.372 |
| *Tobacco use* | 81 | (6.44%) | 59 | (22.69%) | 1,264 | (9.94%) | 45 | (7.77%) | 109 | (14.67%) | 1,584 | (10.14%) | 3,433 | (11.93%) | 0.031 |

*SD: standard deviation, COPD: chronic pulmonary obstructive disease*

*P values were estimated using Chi-square for categorical variables and Anova for continuous variables.*

| **Table S7. Characteristics of baseline initiations for specific short-acting and long-acting drugs.** | | | | | | | | | | | | | | | |
| --- | --- | --- | --- | --- | --- | --- | --- | --- | --- | --- | --- | --- | --- | --- | --- |
|  | **Short-acting** | | | | **Long-acting** | | | | | | | | | |  |
|  | **Morphine** | | **Oxicodone** | | **Fentanyl** | | **Hydromorphone** | | **Morphine** | | **Oxicodone** | | **Tapentadol** | | **p value** |
|  | N=1,258 (82.87%) |  | N=260 (17.13%) |  | N=12,710 (21.75%) |  | N=579 (0.99%) |  | N=743 (1.27%) |  | N=15,617 (26.72%) |  | N=28,787 (49.26%) |  |  |
| Duration, median (IQR) | 8.00 | (4.00, 13.00) | 6.00 | (4.00, 20.00) | 27.00 | (14.00, 59.00) | 29.00 | (27.00, 59.00) | 28.00 | (8.00, 59.00) | 27.00 | (19.00, 55.00) | 29.00 | (14.00, 59.00) | <0.001 |
| Duration strata |  |  |  |  |  |  |  |  |  |  |  |  |  |  |  |
| *1-3 days* | 271 | (21.54%) | 34 | (13.08%) | 306 | (2.41%) | 11 | (1.90%) | 79 | (10.63%) | 379 | (2.43%) | 607 | (2.11%) | <0.001 |
| *4-7 days* | 290 | (23.05%) | 104 | (40.00%) | 627 | (4.93%) | 33 | (5.70%) | 95 | (12.79%) | 1142 | (7.31%) | 2307 | (8.01%) | <0.001 |
| *8-14 days* | 425 | (33.78%) | 36 | (13.85%) | 4218 | (33.19%) | 54 | (9.33%) | 110 | (14.80%) | 2081 | (13.33%) | 4936 | (17.15%) | <0.001 |
| *15-30 days* | 152 | (12.08%) | 46 | (17.69%) | 3190 | (25.10%) | 288 | (49.74%) | 210 | (28.26%) | 6939 | (44.43%) | 11485 | (39.90%) | <0.001 |
| *>30 days* | 120 | (9.54%) | 40 | (15.38%) | 4369 | (34.37%) | 193 | (33.33%) | 249 | (33.51%) | 5076 | (32.50%) | 9452 | (32.83%) | <0.001 |
| Number of prescriptions, mean (SD) | 2.83 | (6.85) | 2.32 | (3.01) | 4.59 | (9.18) | 2.44 | (4.56) | 4.08 | (11.54) | 2.46 | (3.96) | 2.12 | (3.54) | 0.826 |
| Number of prescriptions, range |  |  |  |  |  |  |  |  |  |  |  |  |  |  |  |
| *1* | 731 | (58.11%) | 160 | (61.54%) | 5,044 | (39.69%) | 376 | (64.94%) | 365 | (49.13%) | 9,336 | (59.78%) | 19,740 | (68.57%) | 0.041 |
| *2* | 239 | (19.00%) | 48 | (18.46%) | 3,090 | (24.31%) | 70 | (12.09%) | 134 | (18.03%) | 2,672 | (17.11%) | 3,703 | (12.86%) |  |
| *3 or more* | 288 | (22.89%) | 52 | (20.00%) | 4,576 | (36.00%) | 133 | (22.97%) | 244 | (32.84%) | 3,609 | (23.11%) | 5,344 | (18.56%) |  |
| MME, median (IQR) | 200.00 | (100.00,400.00) | 210.00 | (210.00,630.00) | 1296.00 | (864.00,3456.00) | 960.00 | (480.00,1440.00) | 600.00 | (240.00,1740.00) | 840.00 | (420.00,1260.00) | 1200.00 | (600.00,1800.00) | <0.001 |
| Daily MME, median (IQR) | 21.24 | (11.11,44.44) | 35.00 | (23.66,52.50) | 60.31 | (30.86,78.26) | 32.54 | (16.55,35.68) | 25.71 | (15.79,60.00) | 24.71 | (15.56,44.21) | 41.38 | (20.87,66.67) | <0.001 |
| Daily MME, range |  |  |  |  |  |  |  |  |  |  |  |  |  |  |  |
| *<50* | 954 | (75.83%) | 155 | (59.62%) | 5,748 | (45.22%) | 473 | (81.69%) | 528 | (71.06%) | 12,530 | (80.23%) | 19,013 | (66.05%) | <0.001 |
| *50-89* | 141 | (11.21%) | 62 | (23.85%) | 4,146 | (32.62%) | 47 | (8.12%) | 109 | (14.67%) | 1,617 | (10.35%) | 4,804 | (16.69%) |  |
| *90-119* | 87 | (6.92%) | 24 | (9.23%) | 725 | (5.70%) | 15 | (2.59%) | 18 | (2.42%) | 618 | (3.96%) | 963 | (3.35%) |  |
| *>=120* | 76 | (6.04%) | 19 | (7.31%) | 2,091 | (16.45%) | 44 | (7.60%) | 88 | (11.84%) | 852 | (5.46%) | 4,007 | (13.92%) |  |
| Indication of use |  |  |  |  |  |  |  |  |  |  |  |  |  |  |  |
| *Respiratory* | 243 | (19.32%) | 56 | (21.54%) | 728 | (5.73%) | 9 | (1.55%) | 174 | (23.42%) | 255 | (1.63%) | 449 | (1.56%) | <0.001 |
| *Gastrointestinal pain* | 36 | (2.86%) | 9 | (3.46%) | 425 | (3.34%) | 9 | (1.55%) | 34 | (4.58%) | 407 | (2.61%) | 489 | (1.70%) | <0.001 |
| *Osteoarticular* | 21 | (1.67%) | 22 | (8.46%) | 2,402 | (18.90%) | 183 | (31.61%) | 50 | (6.73%) | 4,094 | (26.22%) | 6,506 | (22.60%) | <0.001 |
| *Back pain* | 16 | (1.27%) | 22 | (8.46%) | 2,643 | (20.79%) | 178 | (30.74%) | 90 | (12.11%) | 5,176 | (33.14%) | 10,664 | (37.04%) | <0.001 |
| *Rheumatoid pain* | 12 | (0.95%) | 9 | (3.46%) | 388 | (3.05%) | 30 | (5.18%) | 14 | (1.88%) | 1,099 | (7.04%) | 2,087 | (7.25%) | <0.001 |
| *Other MSK* | 43 | (3.42%) | 31 | (11.92%) | 1,023 | (8.05%) | 49 | (8.46%) | 44 | (5.92%) | 1,302 | (8.34%) | 2,98 | (10.35%) | <0.001 |
| *Miscellaneous* | 887 | (70.51%) | 111 | (42.69%) | 5,101 | (40.13%) | 121 | (20.90%) | 337 | (45.36%) | 3,284 | (21.03%) | 5,612 | (19.49%) | <0.001 |
| Previous use of medication |  |  |  |  |  |  |  |  |  |  |  |  |  |  |  |
| *NSAIDs or analgesics* | 465 | (36.96%) | 150 | (57.69%) | 7,432 | (58.47%) | 315 | (54.40%) | 335 | (45.09%) | 8,743 | (55.98%) | 15,843 | (55.04%) | <0.001 |
| Overlapping medication |  |  |  |  |  |  |  |  |  |  |  |  |  |  |  |
| *Benzodiazepines* | 480 | (38.16%) | 64 | (24.62%) | 5,198 | (40.90%) | 225 | (38.86%) | 286 | (38.49%) | 5,354 | (34.28%) | 8,895 | (30.90%) | 0.181 |
| *Gabapentinoids* | 30 | (2.38%) | 16 | (6.15%) | 1,065 | (8.38%) | 79 | (13.64%) | 63 | (8.48%) | 1,85 | (11.85%) | 3,392 | (11.78%) | <0.001 |
| *Antipsychotics* | 421 | (33.47%) | 10 | (3.85%) | 1,587 | (12.49%) | 24 | (4.15%) | 71 | (9.56%) | 544 | (3.48%) | 803 | (2.79%) | <0.001 |

*IQR: interquartile range, SD: standard deviation; MME: morphine milligram equivalent; NSAID: Non-steroidal anti-inflammatory drugs, MSK: musculoskeletal*

*P values were estimated using Chi-square for categorical variables and Anova for continuous variables.*

| **Table S8. Characteristics of subsequent initiation, overall and per type of opioid, n (%).** | | | | | | | | | | | | | |
| --- | --- | --- | --- | --- | --- | --- | --- | --- | --- | --- | --- | --- | --- |
|  | Total | | Ultrafast | | Short-acting | | Long-acting | | Tramadol | | Codeine | | P value |
|  | N=552,408 | | N=692(0.13%) | | N=530(0.10%) | | N=51,591(9.34%) | | N=418,194(75.70%) | | N=81,401(14.74%) | |  |
| Duration, median (IQR) | 9.00 | (5.00,29.00) | 13.00 | (5.00,29.00) | 10.00 | (6.00,27.00) | 29.00 | (14.00,62.00) | 9.00 | (6.00,29.00) | 5.00 | (4.00,6.00) | <0.001 |
| Duration strata |  |  |  |  |  |  |  |  |  |  |  |  |  |
| *1-3 days* | 36,299 | (6.57%) | 125 | (18.06%) | 77 | (14.53%) | 1,077 | (2.09%) | 21,278 | (5.09%) | 13,742 | (16.88%) | <0.001 |
| *4-7 days* | 159,579 | (28.89%) | 119 | (17.20%) | 115 | (21.70%) | 3,499 | (6.78%) | 105,981 | (25.34%) | 49,865 | (61.26%) | <0.001 |
| *8-14 days* | 134,22 | (24.30%) | 139 | (20.09%) | 144 | (27.17%) | 8,458 | (16.39%) | 114,366 | (27.35%) | 11,113 | (13.65%) | <0.001 |
| *15-30 days* | 115,806 | (20.96%) | 167 | (24.13%) | 103 | (19.43%) | 18,239 | (35.35%) | 92,809 | (22.19%) | 4,488 | (5.51%) | <0.001 |
| *>360 days* | 106,504 | (19.28%) | 142 | (20.52%) | 91 | (17.17%) | 20,318 | (39.38%) | 83,76 | (20.03%) | 2,193 | (2.69%) | <0.001 |
| Number of prescriptions, mean (SD) | 5.18 | (8.13) | 5.46 | (6.75) | 4.39 | (6.72) | 5.46 | (8.02) | 5.49 | (8.53) | 3.42 | (5.42) | <0.001 |
| Number of prescriptions, range |  |  |  |  |  |  |  |  |  |  |  |  |  |
| *1* | 139,774 | (25.30%) | 173 | (25.00%) | 195 | (36.79%) | 10,912 | (21.15%) | 100,07 | (23.93%) | 28,424 | (34.92%) | <0.001 |
| *2* | 106,142 | (19.21%) | 125 | (18.06%) | 100 | (18.87%) | 9 | (17.44%) | 77,716 | (18.58%) | 19,201 | (23.59%) | <0.001 |
| *3 or more* | 306,492 | (55.48%) | 394 | (56.94%) | 235 | (44.34%) | 31,679 | (61.40%) | 240,408 | (57.49%) | 33,776 | (41.49%) | <0.001 |
| MME, median (IQR) | 150.00 | (90.00,450.00) | 518.70 | (130.00,1111.50) | 210.00 | (100.00,420.00) | 1200.00 | (600.00,2400.00) | 150.00 | (75.00,375.00) | 135.00 | (90.00,135.00) | <0.001 |
| Daily MME |  |  |  |  |  |  |  |  |  |  |  |  |  |
| *Median (IQR)* | 15.52 | (8.33, 25.00) | 35.08 | (17.98, 80.00) | 21.03 | (11.11, 44.44) | 40.45 | (20.69, 63.16) | 12.64 | (8.33, 18.75) | 22.50 | (18.00, 30.00) | <0.001 |
| Mean (SD) | 24.91 | (117.66) | 80.06 | (155.78) | 42.38 | (72.94) | 69.36 | (372.16) | 18.97 | (28.30) | 26.66 | (18.83) | <0.001 |
| Daily MME, range |  |  |  |  |  |  |  |  |  |  |  |  |  |
| *<50* | 508,874 | (92.12%) | 420 | (60.69%) | 410 | (77.36%) | 35,012 | (67.86%) | 396,015 | (94.70%) | 77,017 | (94.61%) | <0.001 |
| *50-89* | 27,499 | (4.98%) | 131 | (18.93%) | 58 | (10.94%) | 8,875 | (17.20%) | 16,082 | (3.85%) | 2,353 | (2.89%) | <0.001 |
| *90-119* | 5,716 | (1.03%) | 30 | (4.34%) | 32 | (6.04%) | 1,932 | (3.74%) | 2,408 | (0.58%) | 1,314 | (1.61%) | <0.001 |
| *>=120* | 10,319 | (1.87%) | 111 | (16.04%) | 30 | (5.66%) | 5,772 | (11.19%) | 3,689 | (0.88%) | 717 | (0.88%) | <0.001 |
| Indication of use |  |  |  |  |  |  |  |  |  |  |  |  |  |
| *Respiratory* | 64,862 | (11.74%) | 47 | (6.79%) | 80 | (15.09%) | 1,861 | (3.61%) | 20,442 | (4.89%) | 42,432 | (52.13%) | <0.001 |
| *Gastrointestinal pain* | 18,312 | (3.31%) | 32 | (4.62%) | 22 | (4.15%) | 1,055 | (2.04%) | 15,095 | (3.61%) | 2,108 | (2.59%) | <0.001 |
| *Osteoarticular* | 113,152 | (20.48%) | 103 | (14.88%) | 51 | (9.62%) | 11,733 | (22.74%) | 94,434 | (22.58%) | 6,831 | (8.39%) | <0.001 |
| *Back pain* | 165,569 | (29.97%) | 109 | (15.75%) | 49 | (9.25%) | 17,644 | (34.20%) | 136,983 | (32.76%) | 10,784 | (13.25%) | <0.001 |
| *Rheumatoid pain* | 42,585 | (7.71%) | 31 | (4.48%) | 5 | (0.94%) | 3,625 | (7.03%) | 35,872 | (8.58%) | 3,052 | (3.75%) | <0.001 |
| *Other MSK* | 52,627 | (9.53%) | 88 | (12.72%) | 30 | (5.66%) | 4,032 | (7.82%) | 43,555 | (10.42%) | 4,922 | (6.05%) | <0.001 |
| *Miscellaneous* | 95,301 | (17.25%) | 282 | (40.75%) | 293 | (55.28%) | 11,641 | (22.56%) | 71,813 | (17.17%) | 11,272 | (13.85%) | <0.001 |
| Previous use of medication |  |  |  |  |  |  |  |  |  |  |  |  |  |
| *NSAIDs or analgesics* | 270,657 | (49.00%) | 369 | (53.32%) | 241 | (45.47%) | 29,132 | (56.47%) | 215,039 | (51.42%) | 25,876 | (31.79%) | <0.001 |
| Overlapping medication |  |  |  |  |  |  |  |  |  |  |  |  |  |
| *Benzodiazepines* | 178,754 | (32.36%) | 263 | (38.01%) | 232 | (43.77%) | 20,66 | (40.05%) | 138,616 | (33.15%) | 18,983 | (23.32%) | <0.001 |
| *Gabapentinoids* | 34,879 | (6.31%) | 91 | (13.15%) | 35 | (6.60%) | 6,235 | (12.09%) | 26,018 | (6.22%) | 2,5 | (3.07%) | <0.001 |
| *Antipsychotics* | 15,417 | (2.79%) | 53 | (7.66%) | 135 | (25.47%) | 2,107 | (4.08%) | 11,36 | (2.72%) | 1,762 | (2.16%) | <0.001 |

*IQR: interquartile range, SD: standard deviation; MME: morphine milligram equivalent; NSAID: Non-steroidal anti-inflammatory drugs, MSK: musculoskeletal*

*P values were estimated using Chi-square for categorical variables and Anova for continuous variables.*
